# Serum NLRP3 Inflammasome and BDNF: Potential Biomarkers Differentiating Reactive and Endogenous Depression

**DOI:** 10.3389/fpsyt.2022.814828

**Published:** 2022-02-28

**Authors:** Xin-Jing Yang, Bing-Cong Zhao, Jing Li, Chuan Shi, Yu-Qing Song, Xing-Zhou Gao, Hui-Li Jiang, Qiu-Yun Yu, Xing-Chen Liang, Shi-Xing Feng, Xiang Li, Yang Sun, Ya-Huan Li, Yang-Peng Wang, Tuya Bao, Zhang-Jin Zhang

**Affiliations:** ^1^Department of Traditional Chinese Medicine, South China Hospital of Shenzhen University, Shenzhen, China; ^2^School of Chinese Medicine, Li Ka Shing Faculty of Medicine, The University of Hong Kong, Hong Kong, Hong Kong SAR, China; ^3^Beijing Key Laboratory of Acupuncture Neuromodulation, Acupuncture and Moxibustion Department, Beijing Hospital of Traditional Chinese Medicine, Capital Medical University, Beijing, China; ^4^Center on Aging Psychology Institute of Psychology, Chinese Academy of Sciences, Beijing, China; ^5^Psychological Assessment Center, Peking University Sixth Hospital, Beijing, China; ^6^Institute of Mental Health, Peking University Sixth Hospital, Beijing, China; ^7^School of Acupuncture-Moxibustion and Tuina, Beijing University of Chinese Medicine, Beijing, China; ^8^The First Affiliated Hospital of Guangzhou University of Chinese Medicine, Guangzhou, China; ^9^Bei Tai Ping Zhuang Community Health Service Center, Beijing, China; ^10^Dongfang Hospital of Beijing University of Chinese Medicine, Beijing, China; ^11^College of Management, Shenzhen University, Shenzhen, China

**Keywords:** mild-to-moderate depression, reactive depression, endogenous depression, NOD-like receptor family protein 3 (NLRP3), brain-derived neurotrophic factor (BDNF)

## Abstract

**Background:**

The highly heterogeneous pathogenesis of depression and limited response to current antidepressants call for more objective evidence for depression subtypes. Reactive and endogenous depression are two etiologically distinct subtypes associated with different treatment responses. This study aims to explore the potential biomarkers that differentiate reactive and endogenous depressions.

**Methods:**

The clinical manifestations and biological indicators of 64 unmedicated mild-to-moderate depression patients (32 reactive depression patients and 32 endogenous depression patients) and 21 healthy subjects were observed. The 24-item Hamilton rating scale for depression (HAMD-24) was used to evaluate the severity of depression. Serum levels of depression-related biological indicators were measured by using the enzyme-linked immunosorbent assay.

**Results:**

The NLRP3 level of reactive depression was significantly lower than those of endogenous depression and healthy controls. There was a significant negative correlation between the BDNF level and the HAMD-24 total scores for patients with reactive depression.

**Conclusion:**

Our findings suggested the serum NLRP3 and BDNF levels could be potential biomarkers for detecting and evaluating the severity of reactive depression.

## Introduction

Depression is a common and costly disorder with clinical and etiological heterogeneity (1). Although antidepressants are the first-line treatment for depression, antidepressant response varies considerably among individuals, ~50–60% of the patients have not achieved adequate response following antidepressant treatment (2). Clinicians have long intuited that heterogeneity in treatment response is the direct result of etiological heterogeneity in depression (3), and treatment should be made more sophisticated by identifying which subtype the depressive episode belongs to (4).

According to the presence or absence of stress before the onset of depression, two etiologically distinct subtypes are categorized as “reactive” (occurring as the result of a stressor) and “endogenous” (occurring in the absence of stress). Reactive depression refers to an inappropriate state of depression precipitated by events or other environmental factors arising as a consequence of severe life events in the person's life. Endogenous depression is a type of depression caused by genetic, somatic, or biological factors rather than environmental influences, in contrast to reactive depression (5, 6). It has been suggested that “endogenous” and “reactive” subtypes of depression are associated with largely distinct biological mechanisms, which respond differentially to antidepressants treatment (3).

Many factors play roles in the development of depression. The role of immune-inflammation in depression has been focused on by researchers in recent years (7). Immunological mechanisms are increasingly implicated in the pathogenesis of depressive symptoms (8). Activation of the peripheral immune system has been consistently associated with depression (9). Immune system-related biological indicators may serve as a valid target for antidepressant treatment (10). Mounting evidence has suggested that the TLR4/NLRP3 signaling pathways may be the new targets for the development and treatments for depression. The NOD-like receptor family protein 3 (NLRP3) inflammasome complex displays a critical role in the pathogenesis and development of inflammation and immune response (11). Upon activation, NLRP3 leads to the maturation of the pro-inflammatory cytokines pro-IL-1β and pro-IL-18 (12). Oxidative stress is presumed to activate the NLRP3 inflammasome and contribute to neuroinflammation (11, 13). A novel aspect of the immune-inflammation process in the response to stress and depression: the NLRP3 inflammasome is a key molecular mechanism that translates psychological stressful stimuli into inflammatory responses (14).

However, not all patients with depression will be peripherally inflamed to the same extent (15). Besides the newly raised immune-inflammation theory, the hyperactivity of the hypothalamic-pituitary-adrenocortical (HPA)-axis (16), the brain-derived neurotrophic factor (BDNF) deficiency (17), and the dopamine (DA) deficiency have been demonstrated in the occurrence and development of depression (18). But the mechanisms underlying these abnormalities between reactive and endogenous depression subtypes remained unclear.

A deeper understanding of how peripheral biomarkers relate to some of the dimensions of heterogeneity encompassed by different subtypes of depression could be an important step toward mechanistically stratified treatment of depression (19). Specifically, we hypothesize the biological indicators expression of reactive depression may differ from that of endogenous depression in depression-related biological mechanisms including classic HPA-axis, BDNF, DA, and novel inflammatory-immune. In this study, related biological indicators in patients with endogenous and reactive depression were observed, to explore the distinct biological mechanisms between the two subtypes of depression.

## Methods

### Participants

This study was a component of a clinical trial in patients with depression approved by the Institutional Review Board (IRB) of Peking University Sixth Hospital, the Institute of Mental Health (IMH, WHO Mental Health Collaborating Center), and registered in www.chictr.org.cn (ChiCTR-IOR-15007551). All participants had given voluntary, written, informed consent before entering the trial, and the study was conducted according to the Declaration of Helsinki.

A total of 85 participants were included in this study. Sixty-four patients with mild-to-moderate depression and 21 healthy controls were recruited. Recruited depression patients met the following inclusion criteria: (1) met the diagnostic criteria of mild-to-moderate depression according to the International Classification of Diseases 10th Edition (ICD-10, F32), with a score of the 24-item Hamilton Depression Scale (HAMD-24) > 8 and ≤35 (20); (2) confirmed to be diagnosed for the first time and the course of illness is 2 weeks−1 year; (3) age 18–60 years. The exclusion criteria were as follows: (1) were receiving anti-depressant treatment; (2) had other mental diseases; (3) were diagnosed with other serious diseases that needed to be treated; (4) current use of any medication (e.g., corticosteroids, antihistamines, and anti-inflammatory medications) likely to compromise interpretation of adrenocorticotropic hormone (ACTH), cortisol (CORT), corticotropin-releasing hormone (CRH), interleukin-1β (IL-1β), interleukin-6 (IL-6), NLRP3, toll-like receptor 4 (TLR4), BDNF, and DA; (5) were pregnant or breastfeeding; (6) presented or suspected to have suicidal plan or behavior, screened by using the Columbia-Suicide Severity Rating Scale (C-SSRS); (7) had participated in other clinical trials in the previous 8 weeks.

Healthy volunteers (age- and sex-matched with depression patients) were included in the healthy control group when they had: (1) a score of HAMD-24 ≤8; (2) no personal or documented family history of depression, anxiety disorders, or other mental diseases; (3) no drug intake during the two weeks before the blood sampling; (4) being in good physical health.

### Clinical Assessment

Basic demographic data and major life events (MLEs) information of all participants were recorded. MLEs were recorded *via* a semi-structured interview aimed at life events. According to the life events and difficulties (LEDS) evaluation system, provoking agents were determined. Provoking agents are psychosocial stressors defined contextually, occurring in a designated period before illness onset, and thought to be causally related to the disorder (21). Patients with provoking agents were allocated to the reactive depression group; patients without provoking agents were allocated to the endogenous depression group.

The severity of depression symptoms of all participants was assessed by using the HAMD-24. The social support rating scale (SSRS) was used to evaluate the social support levels. In addition, participants' pain feelings were recorded on the visual analog pain scale (VAS) ranging from 0 to 10. The MLEs interviews and all clinical assessments were conducted by two interviewers who had participated in training workshops conducted by professional psychiatrists in the IMH. An internal reliability coefficient (*k*-value) >0.80 was achieved after the completion of training.

### Blood Sample Collection and Elisa Measurement

All participants were asked to fast for 8 h before venous blood sampling. Four milliliters of blood were withdrawn from each subject by venipuncture into an anticoagulant-free vacuum tube between 8:00 and 11:00 a.m. After standing for 1–2 h at room temperature, the blood in the tube was centrifuged at 3,000 rpm for 15 min and serum isolated was kept frozen at −80°C. Serum levels of adrenocorticotropic hormone (ACTH) (Cat No.: E01A0005), cortisol (CORT) (Cat No.: E010008), corticotropin-releasing hormone (CRH) (Cat No.: E01P0031), interleukin-1β (IL-1β) (Cat No.: E01I0010), interleukin-6 (IL-6) (Cat No.: E01I0006), NLRP3 (Cat No.: E01N0593), toll-like receptor 4 (TLR4) (Cat No.: E0T0069), BDNF (Cat No.: E01B0029), and DA (Cat No.: E01D0043) were measured by using enzyme-linked immunosorbent assay (Elisa) kits (Blue Gene Biotech CO., LTD, Shanghai, China) according to the manufacturer's recommendations. The ELISA procedures were as follows: leaving the ELISA kits and samples at room temperature (20–25°C) for 30 min; adding 100 μL standards and 100 μL samples to the appropriate well in the antibody pre-coated microtiter plate, and adding 100 μL PBS (pH 7.0–7.2) in the blank control well; dispensing 10 μL balance solution only into 100 μL samples and mixing well; adding 50 μL conjugate to each well; then covering and incubate the plate for 1 h at 37°C; after washing the microtiter plate 5 times and drying the plate, adding 50 μL substrate A and 50 μL substrate B to each well, respectively; covering and incubating for 10–15 min at 37°C on the shaker; adding 50 μL stop solution to each well to stop the reaction; finally, determining the optical density (O.D.) by using MULTISKAN MK3 (Thermo, USA). ELISA was done in duplicates and the intra- and inter-assay coefficients of variation were found to be within 9%.

### Statistical Analysis

Data was entered in the EPI database (Version 3.1) and analyzed with the SPSS Software (IBM. Version 25.0). One-way ANOVA (Least significant difference for *post-hoc* tests) and chi-square (χ^2^) analyses were used to compare sociodemographic and clinical variables among groups. Non-parametric tests were used in the cases where data were not normally distributed. To test the differences in biological indicators' expression levels among groups, the multivariate analysis of covariance (MANCOVA) was conducted; biological indicators were included as the dependent variables; the three groups of subjects were included as the independent variables. The variables gender, age, education, BMI, SSRS, pain, and disease duration were also included in models as covariates to statistically control for the demographic differences among groups. Pairwise comparisons were based on the estimated marginal means, and the least significant difference was used in the adjustment for multiple comparisons. Multivariable regression analysis was used to test whether peripheral biological indicators levels were associated with clinical severity of depression in different groups. The regression model was fit by using the stepwise method. Correlation coefficients between biological indicators levels and HAMD-24 scores were also calculated. Skewed variables were log10 transformed. For log10 transformed data, differences derived from the MANCOVA model for each group were back-transformed. *P* ≤ 0.05 (two-tailed) was considered as statistical significance. Effect sizes on pairwise comparisons were presented as partial eta squared ($\eta_{p}^{2}$) (22).

## Results

### Demographics and Clinical Features

The demographical and clinical features of each group are given in Table 1. The three groups did not have any significant difference in demographic characteristics in terms of gender, age, education, and BMI (*P* > 0.05). There were significant differences among the three groups in HAMD-24 total scores, SSRS scores, and Pain. As expected, *post-hoc* tests indicated that both the two depression groups differed significantly from the healthy control group (*P* < 0.01), and there was no significant difference between the two depression groups in disease duration, HAMD-24 total scores, SSRS scores, and Pain (*P* > 0.05). MLEs for patients in the reactive depression group are classified in Table 1. All events valence was negative and event significance was relatively high according to the standardized list of affect-related life events (23).

**Table 1:** Demographic and clinical characteristics of participants.

**Variables**	**Reactive Depression (*n* = 32)**	**Endogenous depression (*n* = 32)**	**Healthy control (*n* = 21)**	**Group test**	
				**Statistic**	***P*-value**
Gender, female *n* (%)	21 (65.63%)	21 (65.63%)	12 (57.14%)	χ^2^ = 0.49	0.78
Age, years^a^	34.84 (11.20)	37.38 (12.33)	30.95 (5.28)	F = 2.34	0.10
Education, years^b^	16 (2)	16 (5)	18 (6)	H = 5.39	0.07
BMI, kg/m^2a^	22.66 (3.83)	21.35 (2.28)	23.01 (3.29)	F = 2.14	0.12
SSRS^a^	27.69 (8.35)	28.50 (6.98)	34.33 (7.78)^*^^#^	F = 5.26	0.007
Pain^b^ (VAS)	1.5 (4)	2 (5)	1.5 (4)^*^^#^	H = 13.68	0.001
Disease duration, months^b^	7.5 (9)	4.5 (7)	–	*z* = −0.17	0.08
Depression severity^a^ (HAMD-24 total scores)	23.63 (5.96)	22.94 (5.72)	1.90 (1.97)^*^^#^	*F* = 135.21	<0.001
**Major life event in RD group** ***n*** **(%)**					
Serious illness or death of a loved one				7 (18.92%)	
Problems in family or marital relationships				15 (40.54%)	
Problems at work or school				14 (37.84%)	
Others				1 (2.70%)	

a *Normal distribution, expressed as mean (SD)*;

b *Non-normal distribution, expressed as median (IR)*;

* *P < 0.01 vs. RD*;

# *P < 0.01 vs. ED*.

*χ^2^, chi-square test; F, one-way analysis of variance; H, Kruskal–Wallis test; z, Mann–Whitney test; BMI, body mass index; SSRS, social support rating scale; VAS, visual analog pain scale; HAMD-24, 24-item Hamilton rating scale for depression*.

### Biological Indicators' Levels in Two Subtypes Depression Patients and Healthy Controls

**Figure 1:**
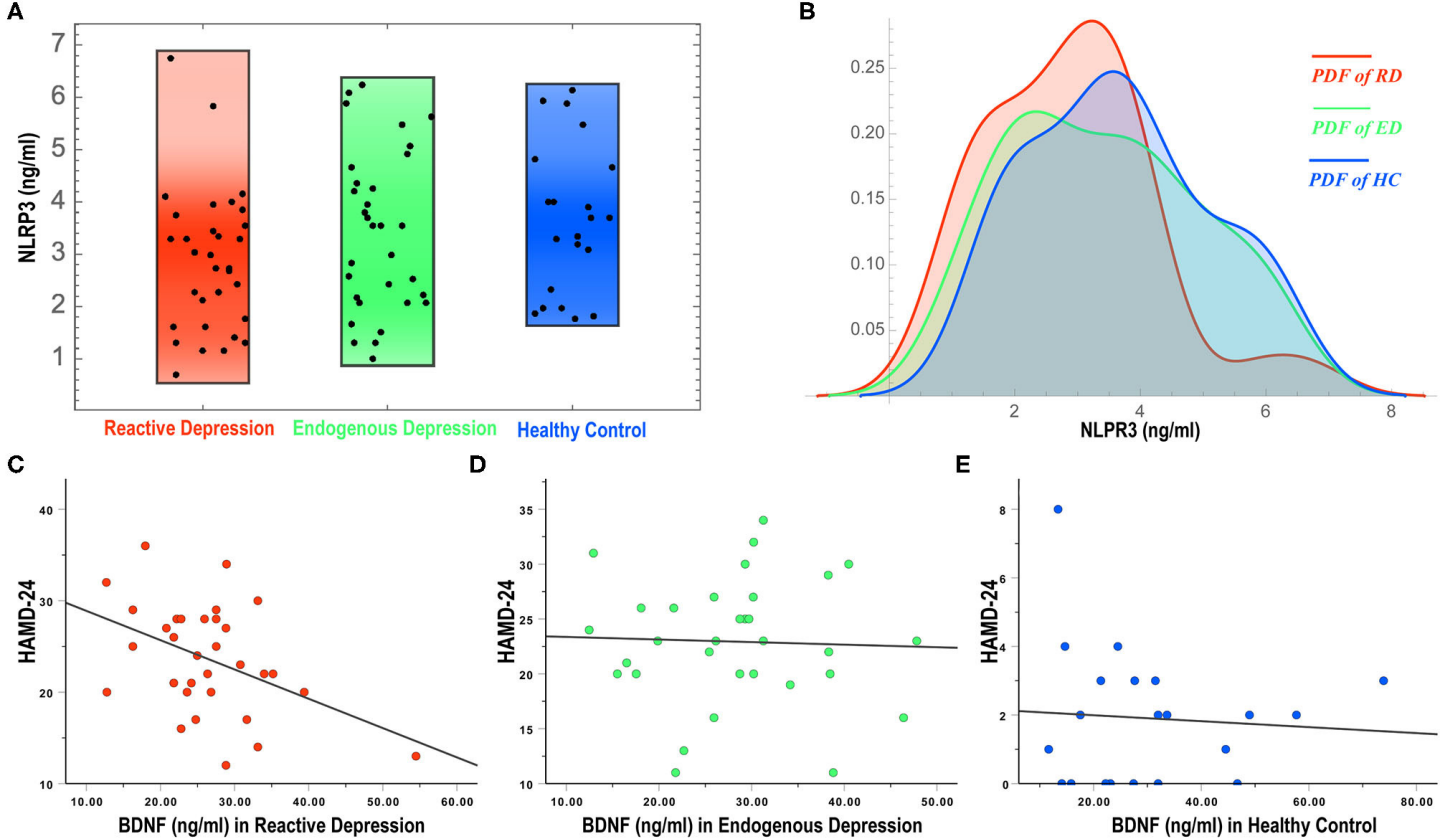
NLRP3 expressions **(A,B)** and correlations of BDNF levels and HAMD-24 **(C–E)** in three groups. NLRP3, NOD-like receptor family protein 3; BDNF, brain-derived neurotrophic factor; RD, reactive depression; ED, endogenous depression; HC, healthy control; PDF, probability density function; HAMD-24, 24-item Hamilton rating scale for depression.

The MANCOVA showed no significant difference in the overall biological indicators among the three groups (Wilks' lambda = 0.707, *F* = 1.411, and *P* = 0.136) with a large effect size (Partial Eta Squared, $\eta_{p}^{2}\;=$ 0.159). Tests of between-subjects effects showed that the NLRP3 expression in the reactive depression group was significantly different among the three groups (*F* = 3.666, *P* = 0.030) and the size of the effect reported medium effect size ($\eta_{p}^{2}$ = 0.089). Pairwise comparisons showed that the expression of NLRP3 in the reactive depression group was significant lower than that of the other two groups (Figures 1A,B and Table 2). Figure 1B shows the probability density functions (PDFs) of the three groups. For each group, its PDF describes the likelihood of the distribution of the corresponding serum NLRP3 level. Significantly, the value of the serum NLRP3 level of the reactive depression group was most likely to occur around 3.2 ng/ml. However, the same serum NLRP3 level began to decrease significantly after reaching about 4 ng/ml, indicating that the serum NLRP3 level of the reactive depression group was significantly less likely to occur between 5 and 8 ng/ml than those of the endogenous depression group and healthy control group.

**Table 2:** Comparison of serum biological indicators expression levels in different groups.

**Variables**	**Reactive depression (*****n*** **=** **32)**		**Endogenous Depression (*****n*** **=** **32)**		**Healthy Control (*****n*** **=** **21)**		**Difference**		
	**Mean**	**95% CI**	**Mean**	**95% CI**	**Mean**	**95% CI**	***F***	***P***	** $\eta_{p}^{2}$ **
ACTH (pg/ml)	67.61	52.48–85.11	72.44	57.54–91.20	47.86	33.88–69.18	1.570	0.215	0.040
CORT (ng/ml)	105.91	93.84–111.97	112.65	101.16–124.15	108.95	91.24–126.66	0.346	0.709	0.009
CRH (pg/ml)	183.94	149.47–218.41	197.41	164.57–230.25	206.43	155.82–257.04	0.272	0.762	0.007
IL-1β (pg/ml)	56.66	46.18–67.15	55.04	45.06–65.03	50.10	34.71–65.50	0.199	0.820	0.005
IL-6 (ng/ml)	158.03	137.71–178.34	139.31	119.95–158.66	134.80	104.97–164.62	1.137	0.326	0.029
NLRP3 (ng /ml)	2.65	2.09–3.20	3.45	2.92–3.98	3.97	3.15–4.78	3.666	0.030	0.089
TLR4 (ng /ml)	5.45	4.45–6.45	4.81	3.86–5.77	4.67	3.19–6.14	0.538	0.586	0.014
BDNF (ng/ml)	25.27	21.07–29.47	28.62	24.62–32.62	31.41	25.25–37.57	1.283	0.283	0.033
DA (ng/ml)	14.03	12.13–15.93	14.76	12.95–16.57	13.66	10.87–16.46	0.272	0.763	0.007

*Adjusted analysis of Multivariate test: Wilks' lambda = 0.707, F = 1.411, p = 0.136, Partial Eta Squared = 0.159. Covariates appearing in the model are evaluated at the following values: sex = 1.64, age = 35.52, edu = 15.91, BMI = 22.2539, SSRS = 29.64, pain = 1.98, and duration = 4.88*.

*CI, confidence interval; $\eta_{p}^{2}$, partial eta squared effect size; ACTH, adrenocorticotropic hormone; CORT, cortisol; CRH, corticotropin-releasing hormone; IL-1β, interleukin-1β; IL-6, interleukin-6; NLRP3, NOD-like receptor family protein 3; TLR4, toll like receptor 4; BDNF, brain-derived neurotrophic factor; DA, dopamine*.

### Correlations of Biological Indicators and the Clinical Severity of Depression

To observe the correlation of biological indicators' levels and the severity of depression, multivariable regression analysis and correlation analysis were conducted. As shown in Table 3, there was a negative correlation between the HAMD-24 scores and the expression levels of BDNF in the reactive depression group (*r* = −0.436, *P* = 0.013) (Figures 1C–E). No correlation between the HAMD-24 scores and the expression levels was observed in the endogenous depression group (*P* > 0.05). For healthy controls, the HAMD-24 scores were positively related with the CORT (*r* = 0.496, *P* = 0.022) and the IL-6 (*r* = 0.436, *P* = 0.048) expression levels.

**Table 3:** Correlations and multivariable regression analyses among serum biological indicators and HAMD-24 total scores in different groups.

**Biological indicators**	**Reactive depression (*****n*** **=** **32)**		**Endogenous Depression (*****n*** **=** **32)**		**Healthy Control (*****n*** **=** **21)**	
	***r*** **^a^**	***P_***c***_***	***r***	***P_***c***_***	***r***	***P_***c***_***
ACTH	−0.238	0.189	0.031	0.868	−0.291	0.200
CORT	−0.338	0.059	0.234	0.197	0.496^*^	0.022
B *=* 0.023, β = 0.399, *P*_r_ = 0.064, *P*_M_ = 4.519						
CRH	0.306	0.089	−0.050	0.786	−0.024	0.918
IL-1β	0.068	0.712	−0.067	0.716	−0.073	0.755
IL-6	0.045	0.808	0.229	0.207	0.436^*^	0.048
B *=* 0.010, β = 0.312, *P*_r_ = 0.140, *P*_M_ = 4.519						
NLRP3	−0.057	0.755	−0.019	0.918	0.128	0.581
TLR4	0.200	0.274	0.180	0.324	0.174	0.452
BDNF	−0.436^*^	0.013	−0.037	0.840	−0.071	0.759
	B *=* −0.320, β = −0.436, *P*_r_ = 0.013, *P*_M_ = 0.013					
DA	0.119	0.518	0.046	0.803	0.418	0.060

* *Denotes statistical significance of the univariate correlation at P < 0.05*;

a *Pearson's r for continuous variables and Spearman's r for variables*.

*t P_c_, P-value of correlation analysis; P_r_, P-value of multivariable regression analysis; P_M_, P-value of Mode. ACTH, adrenocorticotropic hormone; CORT, cortisol; CRH, corticotropin-releasing hormone; IL-1β, interleukin-1β; IL-6, interleukin-6; NLRP3, NOD-like receptor family protein 3; TLR4, toll like receptor 4; BDNF, brain-derived neurotrophic factor; DA, dopamine*.

## Discussion

The main objective of this study was to compare the biological differences between reactive depression and endogenous depression in terms of immune-inflammation, BDNF, DA, and HPA axis-related indicators. Here, we found the patients with reactive depression had specific indicators. The serum NLRP3 level of reactive depression was significantly lower than that of endogenous depression and healthy controls. There was a significant negative correlation between the serum BDNF level of reactive depression and the HAMD-24 total scores.

In combination with vulnerability factors, severe life events are established as provoking agents for depression. Psychological stress plays an important role in the onset of depression characterized by non-endogenous features (21). There is increasing evidence that psychological and physical stressors could activate immune and inflammation processes, contributing to depressive symptoms. Peripheral and central inflammatory responses are relevant to mood regulation (24). NLRP3 is an intracellular multiprotein complex responsible for innate immune processes associated with infection, inflammation, and autoimmunity. NLRP3 activation appears to bridge the gap between immune activation and metabolic danger signals or stress exposure, which are important factors in the pathogenesis of psychiatric disorders (25). Preclinical evidence has proven the link between NLRP3 inflammasome and depressive symptoms (26, 27). Clinical data regarding the involvement of NLRP3 in patients with depression are scarce. Only one report shows that NLRP3 protein levels are increased in the peripheral blood mononuclear cells of patients with depression compared to non-depressed subjects (28). However, it is unknown what is the difference between reactive and endogenous depression in terms of NLRP3 expression. Therefore, in this study, we observed the changes of NLRP3 and its downstream inflammatory cytokines of the two depression subtypes of patients. Notably, we found that the NLRP3 expression level was significantly lower in reactive depression patients compared with endogenous depression patients and healthy controls, while the NLRP3 expression levels of healthy controls and endogenous depression patients were not significantly different. Our result was inconsistent with the previous study. One possible reason is that the subjects observed in this study were patients with mild-to-moderate depression. The pathological mechanism of mild-to-moderate depression is different from that of severe depression. On the other hand, patients with endogenous depression are more likely to have somatic and vegetative symptoms, making endogenous depression more severe than reactive depression. Therefore, biological abnormalities are expected to be more pronounced in the endogenous depression group. However, in this study, the mean HAMD scores of reactive depression patients were higher than those of endogenous depression patients. It may be the reason that the result of this study was inconsistent with that of the previous study.

BDNF is considered to contribute to the nervous system development and function. Chronic stress to genetically vulnerable individuals might induce a significant reduction of BDNF expression (29). Multiple meta-analyses have demonstrated that BDNF is significantly lower in most individuals with untreated depression compared to healthy controls (30). On the other hand, BDNF is sufficient to reduce neuroinflammation (31). Certain antidepressant medications can increase BDNF concentrations in humans (32, 33). In this study, the serum BDNF levels were negatively correlated with the HAMD-24 scores in patients with reactive depression, suggesting that the BDNF levels can reflect the severity of depression in patients with reactive depression. However, we cannot find the correlation between the BDNF levels and the severity of depression in patients with endogenous depression. Similarly, a study found that the acute effect of lamotrigine augmentation therapy for patients with treatment-resistant depressive disorder is not related to BDNF (34), suggesting the BDNF expressions show different change trends with different depressive conditions.

There are several limitations in this study. First, this study was not designed as a diagnostic test. Second, the sample size was relatively small, leading to a limitation for assessing biomarkers. Third, the subjects were limited to patients with mild to moderate depression. The biological differences between reactive depression and endogenous depression of patients with severe depression need to be further studied. Forth, in the statistical analyses, the MANCOVA did not show any significant difference in combined dependent variables among the three groups after controlling for the covariates. Since the *post-hoc* comparisons can reduce the rigorousness of the statistical design, the results and conclusions obtained from these are preliminary and need to be further verified.

In conclusion, NLRP3 is probably a key indicator to differentiate reactive depression from endogenous depression and healthy control. In reactive depression patients, the expression levels of BDNF may reflect the severity of depression. Hence, the serum NLRP3 and BDNF levels could be potential biomarkers for detecting and evaluating the severity of reactive depression. This study represents an important step in the personalization of antidepressant therapy and provides promise for future development and elaboration of biological biomarkers identifying depression of different subtype patients who may be uniquely responsive to different therapies. Further work should examine the utility and possible clinical usefulness of these biomarkers.

## Data Availability Statement

The original contributions presented in the study are included in the article, further inquiries can be directed to the corresponding authors.

## Ethics Statement

The studies involving human participants were reviewed and approved by the Institutional Review Board (IRB) of Peking University Sixth Hospital. The patients/participants provided their written informed consent to participate in this study.

## Author Contributions

TB and Z-JZ: conceptualization and methodology. X-JY and B-CZ: investigation, data curation, formal analysis, and writing—original draft preparation. CS and Y-QS: project administration and patient diagnosis. JL, X-ZG, and Y-PW: investigation and writing—review and editing. H-LJ, Q-YY, X-CL, S-XF, XL, YS, and Y-HL: sample collection and processing. All authors contributed, reviewed, and approved the final manuscript.

## Funding

This study was supported by grants from the National Natural Science Foundation of China (Nos. 81574070 and 82004445).

## Conflict of Interest

The authors declare that the research was conducted in the absence of any commercial or financial relationships that could be construed as a potential conflict of interest.

## Publisher's Note

All claims expressed in this article are solely those of the authors and do not necessarily represent those of their affiliated organizations, or those of the publisher, the editors and the reviewers. Any product that may be evaluated in this article, or claim that may be made by its manufacturer, is not guaranteed or endorsed by the publisher.
